# The challenges of boundary spanners in supporting inter-organizational collaboration in primary care – a qualitative study of general practitioners in a new role

**DOI:** 10.1186/s12875-015-0231-z

**Published:** 2015-02-13

**Authors:** Marius Brostrøm Kousgaard, Anne Sofie Kjær Joensen, Thorkil Thorsen

**Affiliations:** The Research Unit for General Practice and Section of General Practice, Department of Public Health, Faculty of Health Sciences, University of Copenhagen, Øster Farimagsgade 5, PO Box 2099, Copenhagen, DK-1014 Denmark

**Keywords:** Boundary spanners, Denmark, Family practice, General practitioners, Inter-sectoral collaboration, Municipalities

## Abstract

**Background:**

The visions of more integrated care have created new roles and accountabilities for organizations and professionals. Thus, professionals are increasingly expected to engage in boundary spanning activities in order to facilitate inter-organizational and inter-sectoral collaboration. However, this task can be difficult for individual actors and it is important to investigate the work and challenges of boundary spanners in various settings. This study explores the challenges related to a new boundary spanning role for general practitioners employed to facilitate collaboration between the municipalities and general practice.

**Methods:**

The study is based on semi-structured interviews with ten general practitioners acting as municipal practice consultants in the Capital Region of Denmark. The transcribed interviews were analyzed in several steps organizing the material into a set of coherent and distinct categories covering the different types of challenges experienced by the informants.

**Results:**

The main challenges of the general practitioners acting as boundary spanners were: 1) defining and negotiating the role in terms of tasks and competencies; 2) representing and mobilizing colleagues in general practice; 3) navigating in an unfamiliar organizational context.

**Conclusions:**

The results support previous studies in emphasizing the difficult and multifaceted character of the boundary spanning role. While some of these challenges are not easily dealt with due to their structural causes, organizations employing boundary spanners should take note of these challenges and support their boundary spanners with matching resources and competencies.

## Background

Internationally, the increased focus on creating integrated care across and within the different sectors of health care has led to a range of structural changes and created several new roles, tasks, and accountabilities for organizations and professionals [1-6]. In Denmark, the structural reform of 2007 changed the division of work between national, regional and local levels of government [7-9]. One objective of the reform was to improve coordination on administrative and functional levels across different sectors in the health care system with the overall aim of improving conditions for coordinated patient pathways. Notably, local municipalities were given increased responsibilities for providing general and patient specific health prevention as well as rehabilitation services. These new responsibilities for health service delivery have amplified the need for collaboration between the municipalities and general practice [10,11]. To deal with this challenge, several municipalities have created a new job position: the municipal practice consultant (MPC). The MPC is a local general practitioner (GP) hired part time by the municipality with the task of improving collaboration between general practice and the municipal health (and social) agencies. Thus, the MPCs can be characterized as a form of boundary spanners, i.e. “…individuals who have a dedicated job role or responsibility to work in multiorganisational/multisectoral settings, and ‘sustain a connection’… between different interests and practices” [12] p.32. Within the context of public sector management, the notion of boundary spanners has been put forward by Paul Williams in order to highlight the role of individual actors in handling relationships between different organizations [12-15]. According to Williams, the literature on inter-organizational and inter-sectional collaboration has been dominated by a focus on the macro level aspects of collaboration and hence neglected the role of individual actors even though some of these actors may play a significant part in shaping inter-organizational and inter-sectional relations [12]. However, since the task of facilitating collaboration across institutional boundaries can be difficult for individual actors, it is important to explore the work, significance and challenges of boundary spanners in various settings [12,16,17]. The employment of MPCs is a relatively recent phenomenon and there is very limited knowledge about the actual role of the MPCs. Therefore, this study focuses on the early experiences of these GPs hired to engage in boundary spanning activities. More specifically, the aim of this paper is to explore the challenges encountered by GPs in their new role.

### The setting of collaboration

The 98 Danish municipalities are responsible for delivering a range of local welfare services, including some health services such as home nursing and rehabilitation programs for patients with chronic diseases [18]. The general practice sector is composed of approx. 2200 clinics owned and operated by general practitioners (GPs) throughout the country [19]. The GP-clinics are not regulated by the municipalities but via national law and the collective agreement between the Organization of General Practitioners and the Danish Regions responsible for the public funding of GP services. A GP clinic usually receives almost all of its income from public funds. The GPs are reimbursed partly by per-capita payment (from patients listed with the GP) and partly by fee-for-service payment (with the latter constituting about two-thirds of the total income) [19].

In order to facilitate collaboration with general practice, many municipalities have created a new job position – the municipal practice consultant (MPC). The MPC is a general practitioner (GP) who is hired as a consultant by the municipality for a limited number of hours every month. Reimbursement of the MPCs is based on the terms of the collective agreement regarding the use of GPs as external advisors. The MPC has his/her primary work as a GP in a privately owned clinic located in the municipality. General guidelines regarding the conditions of employment and the wage rates for MPCs do exist, but there is a good deal of leverage for local variation on issues such as the number of monthly hours, primary collaborators in the municipality, scope and types of tasks performed by the consultant etc.

Apart from the MPCs, there are two other main types of local institutions that may support collaboration between GPs and the municipality: Local medical guilds and municipal-practice-committees. Local medical guilds are unions of GPs whose clinics are located in the same municipality. These guilds may vary in relation to levels of formality and attendance as well as frequency and content of meetings. Municipal-practice-committees comprise GPs and representatives from the municipality. These committees have an advisory function. The number and composition of members as well as the frequency and content of the meetings vary across municipalities. The role of the MPCs at meetings in the local medical guild or in the municipal-GP-committee is not formally defined and may therefore vary across localities.

## Methods

The study is based on semi-structured interviews with ten municipal practice consultants (MPCs) from the Capital Region of Denmark.

### Selection of informants

A previous survey [20] in the 29 municipalities in the Capital Region of Denmark formed the starting point for selecting MPCs to participate in semi-structured interviews. The survey provided data on issues such as the number of MPCs, their date of employment, the number of consultancy hours per month, the number of local GPs, and the existence of local structures for collaboration such as a municipal-practice-committee and a local medical guild. Among the 18 municipalities that reported having one or more practice consultants, we identified municipalities displaying different characteristics on the variables mentioned above. This strategy of maximum variation sampling [21,22] was aimed at identifying variations in the experiences of practice consultants situated in different local contexts. At the same time, this strategy provides a background against which to identify common patterns that cut across variations [21,23]. The characteristics of the informants are shown in Table 1.

**Table 1:** **Characteristics of informants**

**Interviewee**	**MPC since**	**Number of monthly hours**	**Other local consultants (monthly hours)**	**Number of GPs in municipality (approx.)**	**Local medical guild**	**Local GP-municipality committee**
MPC1	2010	4	No	20	Yes	No
MPC2	2008	8	No	35	Yes	Yes
MPC3	2009	9	Yes (9)	25	Yes	Yes
MPC4	2008	12-14	Yes (6)	70	Yes	Yes
MPC5	2009	16	No	45	Yes	Yes
MPC6	2009	8	No	15	Yes	No
MPC7	2007	1-2	No	30	No	Yes
MPC8	2008	4	No	22	Yes	Yes
MPC9	2010	Ad hoc	No	20	Yes	No
MPC10	2010	12	No	40	Yes	Yes

### Qualitative interviews

A total of ten semi-structured individual interviews with MPCs were carried out between September 2010 and February 2011. The interview guide was designed on the basis of preliminary open interviews with three key informants. These key informants were GPs who originally contributed to shape and promote the MPC as a new position in the municipalities. The interview guide included the following topics: Task definition (who and how), types of tasks performed, the role of the MPC in the existing structures of collaboration formal meetings (e.g. at meetings between the municipality and representatives from general practice), the closest collaborators of the MPC in the municipality, the pre-conceptions and expectations of the municipality, the relationship with local GPs (their expectations and responses to the new role), relationships with other MPCs in or outside the municipality, the challenges of the MPC role, possible dilemmas of boundary spanning role (representation and identification).

The duration of the interviews was 45–70 minutes. Interviews were recorded and transcribed verbatim throughout the data collection process, which contributed to drawing our attention to analytically interesting issues and to make slight adjustments to the interview guide [24]. Also, transcribing interviews as they were completed gave a good overview of the data and provided a background for continuously evaluating whether data saturation was reached, i.e. that no substantially new information was added compared to the previous interviews [22]. As data saturation was assessed to have been achieved, all transcriptions were reviewed to form an overall impression of the material. Using a thematic analytical approach [25], the material was coded and re-coded in the second and third readings, to form an initial list of categories around which to organize the data. Some of these categories corresponded directly to the themes in the interview guide, while others constituted additional categories. For instance, all descriptions of challenges experienced by practice consultants were first coded in one common category ‘challenges’, while in subsequent readings, such broad codes were refined and organized into a set of coherent and distinct categories containing the different types of challenges [26]. In consecutive readings of the material, codes and data were checked against each other to ensure correspondence. Specifically, it was checked whether all analytically interesting aspects of the material were covered in the codes and whether the coding of units of text made sense in the context in which the units appeared in the data material [21].

Formal ethical approval by the research ethics committee system was not required according to Danish law due to the nature of the study (i.e. a non-trial interview study that did not include biomedical material or biomedical data). All informants gave their consent to record the interview and use the material for research publication. The participants were ensured anonymity in the reporting of findings. All authors are trained in the social sciences (MBK and ASJ are political scientists and TT is a sociologist).

## Results

Our analysis identified three main categories of challenges related to the work of the municipal practice consultants. The categories are summarized in Table 2, and below we elaborate on each of the categories.

**Table 2:** **Challenges experienced by practice consultants**

	**Category**	**Description**
1	Defining and negotiating the new role	Lack of clarity and occasional disagreements concerning what specific tasks to take on.
2	Representing and mobilizing colleagues	Lack of a formal mandate to speak and act on behalf of colleagues. Difficulties with engaging colleagues in municipal agendas and activities.
3	Navigating in an unfamiliar organizational context	Insufficient knowledge of the regulatory frame of collaboration. Unfamiliarity with the structure and culture of the municipal organization.

### Defining and negotiating a new role

Nine of the ten municipalities in this study had a formal description of the tasks which the MPC were expected to take on. Common to these descriptions were their overall designation of the MPC as a ‘connecting link’ between the municipality and the local GPs. One description states:

“Collaboration between general practice and the municipality is partly based on the exchange of information about facts such as existing services and organization of work in the municipality, but it also involves catching problems and searching for common solutions”.

Another description includes a list of issues that the MPC may attend to, i.e. distributing new information to general practice, participating in inter-professional health projects, contributing to the development of formal patient pathways or IT-communication tools.

The MPCs expressed rather similar perceptions of the overall purpose of their work, i.e. to act as a link between the municipality and general practice – but from the viewpoint of a GP. There was general agreement among the MPCs that it was not part of their role to get involved in individual patient cases. Rather, MPCs should be concerned with “…shaping the foundations of collaboration” [MPC1]. Most of the MPCs also saw it as an important part of their role to act as information gatekeepers in order to ensure that the GPs are only provided with information directly relevant to their daily work:

“If the municipality wants to say something to the GPs, they tell me and then I pass it on… because they [GPs] are afraid to drown in the stream of information… I simply prioritize what is relevant for the daily work.” [MPC8]

While the MPCs generally agreed on the overall purpose of their role, there was some variation across municipalities concerning the tasks actually performed by the MPCs. Tasks differed from reacting to municipal initiatives and participating in municipal meetings to organizing and summarizing meetings attended by local GPs and administrators, writing newsletters to local GPs and actively seeking to implement joint decisions made in the municipal-practice-committee. It also varied what types of municipal services the MPCs were involved in, e.g. children, elderly, municipal health center or the rehabilitation unit.

In spite of the general descriptions mentioned above, the MPCs found that there were no clear descriptions of specific relevant tasks at the outset of their employment, and most of them considered it difficult to identify such tasks:

‘I experience that it is quite frustrating that my job is so undefined, there are no clear expectations regarding what I should do… I find it difficult to head out on my own to design my job – especially as I don’t really have any political experience’ [MPC1]

Further, the MPCs rarely received suggestions from local GPs regarding what activities to engage in. They tried to deal with this challenge by actively encouraging such suggestions via emails or at meetings in the local medical guild, with the latter being the most useful venue for exploring relevant problems and tasks:

“…I wanted to look at procedures regarding patients living in municipal elderly homes… I had a meeting with the municipality and before that I brought up the topic in the local medical guild [asking] ‘what problems do we have? Do we have any problems with cooperating with the elderly homes?’… I think the medical guild is useful in that way… they [local GPs] want to be asked for advice.” [MPC9]

Another MPC explained that she tried to include her colleagues in the definition of relevant tasks but only received negative replies:

“[When I just started] I lacked someone to discuss with and seek advice from… I only received angry emails from my colleagues with questions such as: ‘why have you not done like this…’ and ‘do not accept any suggestions [from the municipality] before you have asked us’… I wish they had some positive expectations… it is difficult to find out what their needs are, because no one makes suggestions… they are very worried that I promise something that gives them more work or something which is not written in the collective agreement…” [MPC4]

While some MPCs experienced a lack of interest from colleagues in defining relevant tasks, the municipalities did have some expectations or requests. Here, task definition appeared to be a continuous process where tasks were specified in an ad hoc dialogue with the municipal health manager or other municipal employees. However, the ideas and expectations of the municipalities did not always fit with the MPCs’ own understanding of their role. Accordingly, several MPCs reported some challenges in respect to demarcating their role in response to municipal requests. Thus, the MPCs sometimes rejected to take on tasks which they deemed to be irrelevant or inappropriate to engage in:

“[Sometimes I] turn down the municipality. We are rather free as GPs – you know, they cannot fire us… well, they can fire me from the position as a municipal practice consultant but they cannot throw me out of the window in my own clinic, so I think we are pretty strong, and I take advantage of that to share my opinion.” [MPC10]

Another MPC handled disputes over what he perceived to be unrealistic expectations by pointing to the formal limits set on the MPC position:

“I remind the municipality that we have [general] agreements that need to be followed… [If the municipality asks for extra work] then I just reason with them… They know that I only have four hours a week. So I have to watch my time.” [MPC5]

One of the MPCs recounted that his ideas of the consultancy role and relevant tasks were so divergent from those of the municipality that he often found it impossible to reach any consensus. Particularly, he wished to be more involved in the planning and development of municipal health initiatives than the municipality wanted him to be. He reflected:

“It is probably because the task they see a need to solve is not the task that I think needs to be solved. I think, because I am a doctor and I work with the ill and the old and the impaired, then I think health tasks need to be solved, but they [municipality] think it is the obligations regarding prevention and social services that need to be solved.” [MPC2]

A couple of the MPCs did not see task definition as a particularly challenging aspect of their role neither in relation to the municipalities nor in relation to fellow GPs. One said:

“…what is relevant is what we meet in our everyday work… When I meet barriers in my everyday work that is what I am to work with… and that is my job as an intermediate between municipality and general practice… the problems we experience are very tangible and that is where we start” [MPC6]

For nearly all of the MPCs, the starting point for defining and performing their new role was their professional identity as GPs, and some of the MPCs stressed that they consequently preferred to keep a certain distance to the municipality:

“How much should we as GPs interact with the municipality? I think you need to be careful, because you may very easily become intertwined in this. I think it is important to stay focused on what the agenda is for the GP.” [MPC6]

Similarly, another consultant was very clear that she did not want to become too closely integrated in – and identified with – the municipal organization:

“I do not want to be a person from the municipal organization. I am a GP and that is my point of departure, and then I go and listen to what they tell me, and then I disseminate that information [to my colleagues]… I am here in general practice, and then I am a plug to the municipality, but I am not their [the municipality’s] person.” [MPC7]

### Representing and mobilizing colleagues

Generally, the MPCs considered issues of representation and mobilization to be the most challenging aspect of their role, especially due to the lack of a formal mandate:

“…I am hired by the municipality. I cannot represent my colleagues. I have found that difficult. I have tried to discuss with them [GPs] at the meetings in the local medical guild that in a way I would like to represent them but I am not elected by them. [Rather] the municipality has appointed me… I cannot speak on behalf of the others, what they think or don’t think, I have to go back and ask every time, because I do not have any kind of political mandate.” [MPC9]

This lack of mandate could make it difficult for the MPCs to bring forward new ideas for collaboration between the municipality and the local GPs:

“It is difficult to take initiatives… because I cannot really say that I have got back-up from my colleagues. Then it really has to be an obvious improvement, and that is not always the case …I cannot say ‘all GPs would like this suggestion’ because it is very likely that half of them would say ‘what is this? I have never heard of it’, no matter how much we inform in newsletters […]. The mandate to negotiate [with the municipality on behalf of local GPs] is very limited.” [MPC3]

Further, the great variation between local GPs in terms of motivation and areas of professional interests also made it difficult to engage the majority of the local GPs in municipal issues and to gain broad support for the implementation of new initiatives:

“The primary challenge is to mobilize the GPs… some of them I only see very rarely… and the 8–9 GPs [out of the app. 25 GPs in the municipality] that I often see, well that is fine, but it is not enough. You know it has to be practically all GPs that are OK with the changes made… if you keep running and you discover that all the others are not following then it just does not make a difference. And then you may say it is a dead duck. In that case, it is just collaboration between me and the municipality and not between local GPs and the municipality.” [MPC3]

“I would like to make some agreements but I don’t expect back-up from my colleagues… they do as they want and no one else is to interfere with that… a lack of interest, really, in the importance of collaborating with the municipality.” [MPC1]

The MCPs attempted to handle the challenges of representation and mobilization in several ways; by raising municipal issues at meetings in the medical guild, by communicating with colleagues via newsletters and emails, and by drawing on the knowledge and opinions of local GPs involved in negotiations with the public authorities as formal representatives of the profession.

One MPC explained how he made active use of the local municipal-practice-committee and its GP members to discuss new initiatives before implementation was attempted:

“There are a lot of minor things I can deal with on a daily basis [as a consultant]… and then there may be some more fundamental questions where I use the local municipal-practice-committee and the GP committee members to discuss these questions [e.g. design of communications tools and development of chronic disease management programs][…] if I am to have a backing for the things I agree on with the municipality then it is really nice to bring it up in a committee meeting… because there you can sort of say ‘go for it’.” [MPC5]

This MPC also explained that he had tested a new initiative (regular meetings between GPs and social workers involved in cases of sickness leave) by running a pilot among the GPs in the local municipal-GP committee. In that way, the relevance and practicality of the initiative was checked before all local GPs were invited to participate. Another MPC explained that she used the guidelines from The Danish College of General Practitioners (DCGP) as a supplement to asking her colleagues for input on particular issues:

“There is simply a consensus that I inquire with my colleagues, and of course, as GPs we are all little popes, right, and we have our own things, but we also have some guidelines on which we base our work… I do not necessarily answer on behalf of all the local GPs… I cannot do that, but of course I use myself and my references […] where I think that it is reasonable in relation to the guidelines from DCGP.” [MPC6]

Other MPCs explained that they checked some questions with their colleagues via emails or by bringing up the subject for discussion in the local medical guild. However, some of the MPCs experienced that their colleagues did not react when asked for input on municipal issues. Some chose to interpret this as silent consent. Thus, if a suggestion had been put forward and no one objected, then the MPC would proceed as he/she saw fit:

“… part of the way, it is […] those who make an effort [who have a say]… so we raise a topic in the local medical guild – whether something is okay – and if nobody objects, then we cannot [keep asking for acceptance]… A few times, I have send emails with information to all local GPs saying ‘this is how things are’, and then you may say that people are informed, and if they don’t say anything that is it.” [MPC3]

One MPC underlined that the implementation of new initiatives depended on GPs perceiving that it made good sense for them to engage. Therefore, it was important to show that new changes were meaningful:

“Fundamentally, it is a problem that I can [only] make some statements of intent. I can say ‘it would be really good if we could make it work like this’, but I cannot oblige my colleagues, when it is not part of the collective agreement…that is the reality. But then you may hope that as time goes by people [GPs] realize – through the good example – that there are some things that are clever to do that way.” [MPC5]

A few of the MPCs found representation and mobilization to be particularly difficult because they had a different take on some issues than their colleagues. One MPC reflected on the (lack of) motivation on the part of her colleagues to participate in municipal meetings:

[MPC1]: “They [the municipality] are not willing to pay the GPs for attending meetings.”

[Interviewer]: “Do you think that would help [on attendance levels]?”

[MPC1]: “I think it would, but personally, I sympathize with the municipal point of view. I think it is an obligation on the part of GPs to participate in such meetings… I definitely think one should be expected to attend without being paid.”

Another consultant with similar experiences had become careful when attempting to speak on behalf of the local group of GPs:

“Some of the things that I personally find simple, my colleagues don’t find simple… And I have become more attentive not to say anything about anything before I have cleared it with [a local GP with political experience] or the rest of the group.” [MPC4]

Despite the challenges identified above, the MPCs generally perceived that their work had a positive impact on the relations between the municipality and general practice in terms of solving some practical problems and improving mutual understanding. However, they found it difficult to document the specific effects of their work.

### Navigating in an unfamiliar organizational context

Some of the MPCs thought that they did not have sufficient knowledge about the formal regulations influencing the relationship between the municipalities and general practice. Especially, they had problems determining the implications of the collective agreement for the division of work between GPs and the municipality. This uncertainty made it difficult to provide a clear response to municipal requests or to solve local disputes over the respective duties and responsibilities of the two parties. Two of the MPCs often leaned on the knowledge of a colleague with more political experience. One of them says:

“I would like a definition of the role, and maybe an introduction to the collective agreement… because I think that has been really hard… because you interpret it in different ways… I have talked a lot to [a local GP with political experience from the regional level]… I mostly send him an email or call him [to clear some things]” [MPC4].

This MPC – like others – also expected to make more use of meetings in the regional group of MPCs to raise questions about the implications of the collective agreement for the division of work between municipalities and GPs.

Before being employed as MPCs, most of the informants only had superficial knowledge of the municipality as a political and administrative organization. Navigating in an organizational structure and culture very different from the one in general practice could occasionally give rise to frustrations with the municipalities’ way of working:

“Sometimes there are too many levels on the way, but that is the municipal structure and I cannot do much about that, but it may frustrate me a bit… it is a heavy machine.” [MPC6]

Other MPCs emphasized how the municipal culture of frequent meetings was something they were not used to from general practice:

“That thing about meetings… I think there is a lot of talking and producing documents… compared to how many people are out in the field. There are incredibly many [people] employed to do… well, I don’t know what they do.” [MPC9]

Some MPCs also experienced that the divisional organizational structure of the municipality made it difficult to implement e.g. integrated care pathways or common IT solutions involving different departments:

“I have realized that when we use the word ‘municipality’ we are hopelessly naive, because there is no such thing as a municipality. [Rather], there are a number of municipal departments” [MPC2]

The challenge of navigating in another organizational context seemed to be most present for MPCs who were still new to the role. The more experienced MPCs considered the political and bureaucratic aspects of the municipalities as basic conditions that they just had to deal with in their work by understanding and explaining these conditions to their colleagues in order to avoid misunderstandings and unrealistic expectations.

## Discussion

The study found that the challenges experienced by general practitioners, working as inter-sectoral boundary spanners between municipalities and general practice, could be divided into three categories: 1) Defining and negotiating the new role; 2) Representing and mobilizing colleagues; 3) Navigating in an unfamiliar organizational context.

The first and (especially) the second of these categories were deemed to be the most difficult for the MPCs. These challenges are closely related to some of the basic properties of the organizational context: First, the field consists of a large number of independent general practices that interact with the municipalities in different ways depending on the diagnosis and situation of the patient as well as on the GP’s knowledge and past experiences of working with the municipal services. This heterogeneity creates difficulties for the MPCs in terms of identifying relevant tasks, gathering an overview of opinions and interests, and articulating a consensus perspective from general practice. Second, the regulative structure of the field means that the municipalities do not have an overall formal authority or legitimacy to define a mandatory agenda of tasks and competences for general practice or vice versa. Such basic structural conditions are not easily dealt with by individuals assigned with the task of improving collaboration [27]. In our case defining relevant problems and developing and implementing acceptable solutions therefore becomes an ongoing process of formal and informal negotiations between the MPC, the local GPs, and the municipality. And since the formal authority of the MPCs is rather limited, the MPCs depend on their social competencies and positioning in the local professional networks. This corresponds to results from previous research stressing the importance of networking and brokering in non-hierarchical inter-organizational environments [12,13].

In the process of articulating specific focus areas and implementing new initiatives, boundary spanners may experience what Williams calls “dilemmas of multiple accountabilities”, i.e. the boundary spanner may feel divided between the diverging perspectives and interests of the various organizations and individuals involved in collaboration [12]. Here, Williams finds that boundary spanners mainly see themselves as accountable to the organization which employs them. However, in the cases studied by Williams, the boundary spanners were employed full-time by one of the involved organizations whereas in our case, boundary spanning is only a part time position held by actors whose primary income and professional identity stem from their clinical practice. So, although a few MPCs had experienced mistrust from colleagues who were skeptical about the municipal agenda and although a few MPCs disagreed with their colleagues on some issues, the MPCs generally seemed to shape their role in accordance with their professional identity as GPs. This is in accordance with the conclusions of Braithwaite [28] that professionals who engage in boundary spanning activities usually maintain a strong professional identification. Further, this point also emphasizes that it is important to be clear on the differences between the formal frames of boundary spanning roles when comparing results from different studies of boundary spanning as an inter-organizational phenomenon.

Finally, the problems with defining the content of the MPC-role suggest that some municipalities may have been attracted to the popular idea of having an MPC without thorough consideration of the exact tasks to be handled. Thus, it is plausible that some degree of organizational imitation [29] have been at work during the spread of the MPC-concept among the municipalities.

Recent institutional changes may augur new times for the MPC role. Thus, the establishment of municipal-practice-committees has become mandatory for all municipalities so that representatives from the municipality and general practice can meet regularly and discuss local matters of collaboration. At the same time, the GPs have become more formally organized in local medical guilds in all municipalities in order to ensure formal representation in the committees. The increased presence and importance of municipal-practice-committees can have various consequences for the MPCs. On the one hand, the MPCs may be rendered superfluous over time as formal meetings between the municipalities and local representatives of general practice will provide a regular and constructive basis for interaction between the parties. On the other hand, the MPC may still have a role to play in the new institutional set-up by contributing with decision input and support in the practical implementation of decisions made in the municipal-practice committees. Here, much will depend on how the funding municipalities assess the worth of the MPCs, since demonstrating value is often a key element in the institutionalization of new professional roles [30].

### Strengths and limitations

The interviews provided rich data on the early experiences of the MPCs and the challenges of acting as boundary spanners between the municipalities and general practice. However, the exploratory nature of the study also imposes certain limitations. Thus, the study does not include other actors such as local GPs or municipal employees whose experiences could have provided different perspectives on the accounts of the MPCs and new information on the perceived usefulness and impact of the boundary spanners.

For practical reasons, the present study was confined to municipalities located in the Capital Region of Denmark. However, we do not have any reason to expect that the types of challenges identified in this study are unique to MPCs in this region. Thus, the findings from this study are most likely transferable to other regions in Denmark and plausible also to other countries where similar boundary spanning roles are considered to facilitate collaboration between numerous small health providers and local health authorities.

## Conclusion

This study has identified some key challenges of boundary spanners attempting to improve inter-sectoral collaboration in primary care. The results corresponds well with earlier work from different settings in emphasizing the difficult and multifaceted character of the boundary spanning role and suggest that the challenges facing boundary spanners in primary care are at least as comprehensive as in other public settings. While some of these challenges are not easily dealt with due to their structural causes, organizations employing boundary spanners should still take note of these challenges and attempt to support their boundary spanners with matching resources and competencies. Furthermore, the specific purpose and required tasks should be considered carefully when setting up a boundary spanning position. Future studies could complement these results by investigating the value of formal boundary spanning positions for improving inter-sectoral collaboration. Such evaluations should include the experiences of closely involved stakeholders such as public managers and local health professionals.

## Abbreviations

GP: General practitioner
MPC: Municipal practice consultant
